# Oxidative Damage, Energy Imbalance, and Behavioral Disruptions in *Girardia tigrina* Exposed to Treated Sewage Effluents

**DOI:** 10.1002/tox.70098

**Published:** 2026-04-08

**Authors:** Renata M. P. Freitas, Pedro H. Carvalho, Daniela de Melo e Silva, Carlos Gravato

**Affiliations:** ^1^ Department of Biology, Faculty of Sciences University of Lisbon Lisbon Portugal; ^2^ Center for Ecology, Evolution and Environmental Change (cE3c) & CHANGE—Institute for Global Change and Sustainability, Faculty of Sciences University of Lisbon Lisbon Portugal; ^3^ Institute of Biological Sciences Federal University of Goiás (UFG) Goiânia Brazil; ^4^ Faculty of Pharmacy Federal University of Goiás (UFG) Goiânia Brazil

**Keywords:** energy metabolism, locomotor activity, oxidative stress, regeneration, urban effluents

## Abstract

This study assessed the ecotoxicological effects of treated sewage on the freshwater planarian *Girardia tigrina* , integrating biochemical, energetic, and behavioral biomarkers. Planarians were exposed for 48 h to different concentrations of treated sewage (12.5%, 25%, 50%, and 100%) and compared to controls using commercial water and water from a preserved area (Flona). Exposure to 100% treated sewage caused 100% mortality within 48 h. At sublethal concentrations, biochemical analyses indicated oxidative stress, with increased lipid peroxidation (LPO) at 12.5% and 25%, reduced total glutathione (TG), and elevated catalase (CAT) activity at 25% and 50%. Energetic metabolism was also affected: aerobic metabolism decreased, while anaerobic metabolism increased at 25% and 50%, indicating a shift in energy production pathways under stress. Behaviorally, planarians exposed to 50% sewage showed reduced locomotor activity, and all concentrations delayed photoreceptor regeneration. Despite pollutant levels being below Brazilian limits, several pesticides exceeded European standards, reinforcing concerns about chronic effects of complex mixtures in treated effluents. Biological responses suggested early signs of accelerated aging, with impaired regeneration and mobility. These higher‐level effects were preceded by biochemical disturbances, including reduced antioxidant capacity and a shift from aerobic to anaerobic energy metabolism. The rise in anaerobic activity highlights extreme stress at concentrations ≥ 25%. Overall, findings demonstrate that even low concentrations of treated sewage containing pesticide mixtures can cause sublethal but significant effects in non‐target organisms. The study underscores the value of planarians as sensitive bioindicators and highlights the urgency of enhancing sewage treatment and regulatory standards to better protect aquatic ecosystems.

## 1 Introduction

Water quality is currently a matter of global concern due to the expansion of anthropogenic activities and climate change that pose serious threats to water resources availability [1, 2]. One of the United Nations (UN) Sustainable Development Goals (SDGs) is to ensure the availability and sustainable management of clean water and sanitation for all by 2030. This goal is interconnected with several other SDGs, including those related to health and well‐being, responsible consumption and production, protection of aquatic and terrestrial life, and climate action [3].

In Brazil, 45% of domestic sewage is not treated [4]. Moreover, even after conventional biological treatment, mixtures of substances such as metals, microplastics, pharmaceuticals, and other classes of compounds often remain in domestic effluents [5, 6]. As a result, those mixtures of pollutants are discharged into lakes and rivers, compromising water quality. Consequently, such contaminants are also detected in surface and groundwater, and may therefore be present in drinking water, soil, and food, posing potentially toxic risks not only to humans but also to other organisms [7].

In this study, treated sewage samples were collected from a sewage treatment plant (STP, Goiânia) located in the urban area of the city of Goiânia, in the state of Goiás, Brazil. During the study period, the STP operated with a chemically assisted primary treatment system, designed to achieve approximately 50% removal of 5‐day biochemical oxygen demand (BOD_5_) and about 80% removal of suspended solids. The treatment process included a liquid phase in which a chemical coagulant (typically based on aluminum or iron salts) and a flocculation aid (polymeric flocculant) were added to promote particle aggregation and sedimentation. These steps enhance the removal of suspended solids and organic matter prior to the settling stage. Following the liquid treatment phase, the generated sludge underwent dewatering through centrifugation and subsequent stabilization with lime to adjust pH and reduce pathogen activity before final disposal. Also, water was collected from a second sampling site that is located in a conservation unit area of the municipality of Silvânia, also in the state of Goiás (Flona, Silvânia). These two areas differ in terms of land use and land cover, degree of anthropogenic impact, and pollution levels. Previous studies conducted by our research group have shown that vertebrates such as birds [8] and bats [9] collected from STP showed important deleterious effects and exposure to metals. The comparison of results shows that animals collected at STP exhibit a higher frequency of DNA damage, increased concentrations of metals in fur and feathers, and a more pronounced inflammatory response than animals collected at Flona conservation area. Exposure to pollutants in these animals occurs through inhalation, dermal contact, and ingestion of contaminated food and water [10, 11].

The metabolic mechanisms that induce genetic damage to the animals exposed to STP are not completely understood yet, but are probably related to exposure to the mixture of pollutants present in sewage effluents discharged into lakes and rivers. Therefore, this research aims to determine in controlled laboratory conditions the effects induced in freshwater planarians exposed to treated sewage collected at STP, by comparison with organisms exposed to water from Flona and commercial water used in the laboratory to maintain planarians in culture.

The freshwater planarians are widely recognized as good indicators of water quality [12, 13]. Their popularity in ecotoxicological studies related to aquatic pollution is due to their ease of maintenance in the laboratory, versatility in the use of different toxicological markers, and the presence of biological systems that share similarities with those of vertebrates. Although planarians are considered sensitive models, their tolerance to chemical stress may differ from other invertebrate taxa, reflecting species‐specific physiological and metabolic traits [12, 13]. In freshwater biomonitoring programs, other macroinvertebrate groups such as Ephemeroptera, Plecoptera, and Trichoptera are often recognized as highly sensitive indicators of environmental quality [14]. Previous research studies indicated increased genotoxicity in freshwater planarians that show greater damage in aquatic environments close to urban areas or nearby industrial effluent discharges [15, 16]. Additionally, planarians exposed to water from polluted rivers showed induction of oxidative stress with damage to lipid membranes, likely due to metallic ions present in the water of rivers [17].

It is hypothesized that the mixture of pesticides present in treated sewage requires more energetic input for the detoxification processes of planarians. Also, that the aerobic production of energy together with detoxification might increase the levels of damage in macromolecules due to insufficient antioxidant capacity. This imbalance leading to the accumulation of reactive oxygen species (ROS) levels can cause oxidative stress damaging DNA, lipids, and proteins. Furthermore, the allocation of energy for detoxification, antioxidant capacity and repair systems will affect other physiological functions at the whole‐organism level, such as locomotor activity and regeneration of planarians. Therefore, this research work investigates the lipid peroxidation (oxidative damage), non–enzymatic and enzymatic antioxidant capacities (total glutathione and catalase, respectively), detoxification process (glutathione S‐transferase as phase II biotransformation activity), aerobic and anaerobic production of energy (electron transport system and lactate dehydrogenase, respectively), neurotoxicity (cholinesterase), as well as locomotor activity and post‐exposure regeneration of photoreceptors in decapitated planarians.

## 2 Materials and Methods

### 2.1 Water Samples

Water samples were collected from two contrasting areas in Goiás State, Brazil (Figure 1). The first site was a sewage treatment plant (STP) located in Goiânia, which treats approximately 70% of the total sewage generated in the city [18]. The municipality has an extensive sewerage network that collects predominantly domestic wastewater from residential areas, although the system may also receive contributions from small commercial establishments and urban runoff. Industrial effluents and large‐scale agricultural effluents are not directly connected to the network. The sewerage system is predominantly separated, with wastewater and rainwater collected through distinct drainage systems, although occasional inflow during heavy rainfall events may occur [18]. At this site, about 2 L of treated effluent was collected directly from the settling tank immediately after the treatment process, using a manual pump.

**FIGURE 1:**
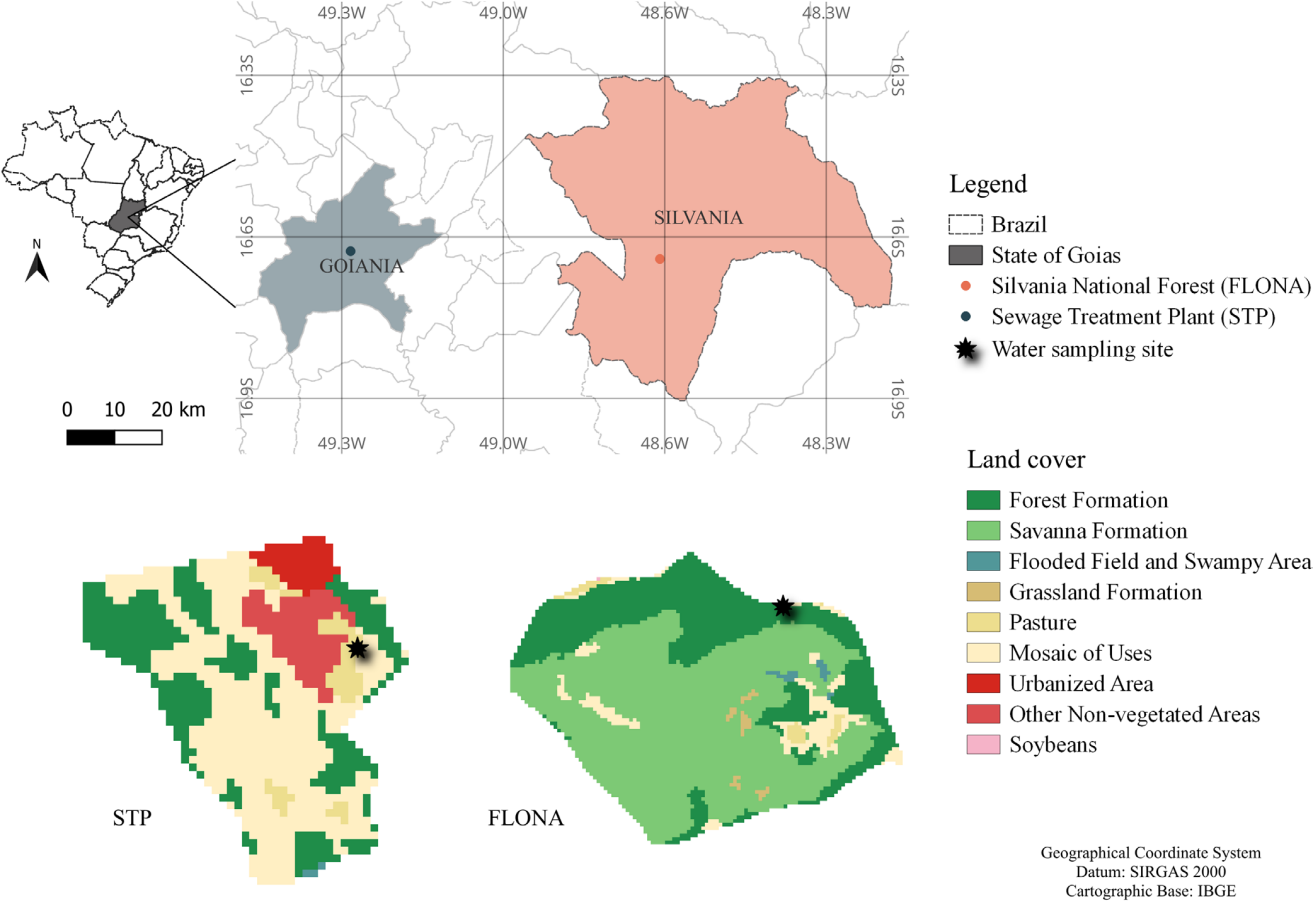
Location of the study area in Goiás State, Brazil, highlighting Silvânia National Forest (FLONA) and the Sewage Treatment Plant (STP). The star indicates the water sampling sites. The lower maps represent the land cover surrounding each sampling location according to land use classification.

The second site was the Silvânia National Forest (Flona), a Conservation Unit in the municipality of Silvânia, selected as a reference area due to its preserved environmental conditions and absence of direct point sources of pollution. At this site, approximately 2 L of water were collected from a lake surrounded by well‐preserved native Cerrado vegetation, using an appropriate container, with sampling performed approximately 2 m from the edge and below the water surface to avoid the collection of the surface layer. The sampling point was located at approximately 16°37′52.9″ S 48°39′08.9″ W. The lake receives water mainly from small natural drainage channels within the forest area and does not receive direct wastewater discharges.

For both sites, sampling points were selected in areas without direct influence from downstream point sources of pollution. All samples were stored in appropriate glass containers and transported to the laboratory under refrigerated conditions until further analyses.

The water samples from both environments were analyzed for inorganic elements (magnesium (Mg), phosphorus (P), potassium (K), chromium (Cr), manganese (Mn), iron (Fe), cobalt (Co), nickel (Ni), copper (Cu), zinc (Zn), arsenic (As), molybdenum (Mo), cadmium (Cd), and lead (Pb)), and pesticide (Acetamiprid, Alachlor, Aldrin, Ametryn, Atrazine, Azinphos‐methyl, Azoxystrobin, Bifenthrin, Carbaryl, Carbendazim, Carbofuran, Diuron, Lambda‐cyhalothrin, Cyfluthrin, Cypermethrin, Cyproconazole, Chlordane, Chlorfenapyr, Chlorfenvinphos, Chlorobenzilate, Chlorpyrifos, Dichlorodiphenyldichloroethane (p,p′‐DDD), Dichlorodiphenyldichloroethylene (p,p′‐DDE), Dichlorodiphenyltrichloroethane (p,p′‐DDT), Deltamethrin, Diazinon, Dicofol, Dicrotophos, Dieldrin, Difenoconazole, Dimethoate, Disulfoton, Dodecachlor, α‐Endosulfan, Endosulfan sulfate, Endrin, Epoxiconazole, Esfenvalerate, Ethion, Fenpropathrin, Fenitrothion, Fenthion, Phenthoate, Fenvalerate, Fipronil, Fipronil sulfone, Folpet, Phorate, Phosalone, Phosmet, Hexachlorobenzene, α‐Hexachlorocyclohexane, β‐Hexachlorocyclohexane, Lindane, Heptachlor, Heptachlor epoxide, Imidacloprid, Iprodione, Malaoxon, Malathion, Methidathion, Metalaxyl, Methomyl, Methoxychlor, Mevinphos, Piperonyl butoxide, Ethyl parathion, Methyl parathion, Permethrin, Pyraclostrobin, Pirimiphos‐methyl, Procymidone, Profenofos, Propargite, Propazine, Propiconazole, Propoxur, Quintozene, Simazine, Tebuconazole, Tetraconazole, Tetradifon, Thiabendazole, Thiacloprid, Thiamethoxam, Trifloxystrobin, Trifluralin) concentrations.

For metal analysis, an aliquot of approximately 8 mL of each sample was digested by adding 2 mL of a mixture of concentrated nitric acid and hydrogen peroxide, in a 1:1 ratio, to a hot plate in a 60°C bath for 4 h. Afterwards, the solution containing the sample was solubilized in 15 mL of a 5% nitric acid solution. Subsequently, the samples were diluted another 1.83 times in 5% nitric acid and then analyzed. Chemical analysis of the elements was performed on a mass spectrometer (Perkin Elmer NexION 350D). Calibration was performed using certified element standards of the analytes of interest (Mg, P, K, Cr, Mn, Fe Co, Ni, Cu, Zn, As, Mo, Cd and Pb) and the element Rhodium (Rh) as an internal standard. Elements with atomic mass less than 80 arbitrary units. were analyzed in collision mode (KED). The analyzed elements are represented in parts per million (ppm) ([19]).

For pesticide analysis, water samples (1 L), with adjusted pH, were sequentially extracted with dichloromethane using a separatory funnel. The extracts were concentrated and transferred to a suitable solvent for analysis. Identification and quantification of the pesticides were performed using gas chromatography coupled with mass spectrometry (GC/MS), with injection into a fused‐silica capillary column and a temperature program optimized for analyte separation. Identification was based on the comparison of retention times and mass spectra with certified analytical standards, and quantification was performed using a calibration curve with an internal standard, according to Methods 3510C and 8270E [20, 21] of the United States Environmental Protection Agency (EPA).

### 2.2 *Girardia tigrina*

Planarians were maintained under controlled conditions (21°C ± 1°C; aeration; darkness) at the Laboratory of Animal Physiology and Planarians (PLANATORY), Faculty of Sciences, University of Lisbon (Portugal), since 2019. Cultures were maintained using commercial water (composition shown in Table 1), which was renewed after feeding the planarians with bovine liver once a week for 5 h under the same conditions. Cultures were maintained with sexual reproduction. Planarians used in the experiments were 4–5 months old, had an average length of approximately 1 cm and an average wet weight of 0.015 g, were deprived of food for 7 days prior to exposure, and were selected based on the absence of apparent anatomical or behavioral damage.

**TABLE 1:** Physicochemical parameters of commercial water (control), treated sewage (STP), and natural water (Flona) used in the exposure of planarians.

Parameters	Commercial water^a^	Treated sewage (STP)^b^	Natural water (Flona)^b^
Total mineralization	39 ± 14 mg/L	—	—
Silica (SiO_2_)	17 ± 5.5 mg/L	—	—
Sodium (Na^+^)	4.4 ± 1.1 mg/L	—	—
Calcium (Ca^2+^)	2.7 ± 1.6 mg/L	—	—
Bicarbonate (HCO_3_^−^)	16.5 ± 8 mg/L	—	—
Chloride (Cl^−^)	3.2 ± 0.9 mg/L	—	—
pH	5.8–7.0	7.3	7.42
Temperature	—	28.66°C	22.41°C
Total dissolved solids (TDS)	—	511 ppm	33 ppm
Dissolved oxygen (DO)	—	64.5%	53.6%
Electrical conductivity	—	1093 μS/cm^2^	63 μS/cm^2^
Turbidity	—	10.4 FNU	74.2 FNU
Oxidation–reduction potential (ORP)	—	296.4 mV	−51.9 mV

^a^ Parameters provided by the manufacturer.

^b^ Parameters measured with a multiparameter probe (Hannah‐HI 9829).

### 2.3 Survival Tests

The survival test was conducted for 48 h with 3 replicates per treatment and 10 organisms per treatment. The exposure groups were 100%, 50%, 25%, and 12.5% of STP effluent, 100% water from Flona, and a control (commercial water only). Effluent dilutions were made with commercial water. The test was conducted in glass containers with 40 mL of each solution. The test was performed under controlled conditions as previously described for cultures of planarians. Planarians were not fed during the survival test. Mortality was checked every 12 h until the end of exposure, and the number of dead organisms was recorded for each replicate of each treatment. All planarians in the group exposed to 100% treated sewage died within 48 h of the experiment. No mortality was observed for the other groups.

Part of the organisms (*n* = 10 per treatment) of the experimental groups that survived the survival tests (12.5%, 25%, and 50% STP effluent, 100% Flona water, and the control using commercial water) were sampled, weighed and individually stored at −80°C until used for biochemical.

### 2.4 Biomarkers

#### 2.4.1 Sample Preparation

Eight stainless steel beads and 1 mL of ultrapure water were added to each tube containing one planarian and. Samples were homogenized for 2 + 2 min at 30 Hz frequency. Then, another 1 mL of ultrapure water were added to each tube. The homogenate of each sample was divided in eight microtubes that were used to evaluate lipid peroxidation (LPO), total glutathione (TG) and protein levels, as well as activities of catalase (CAT), glutathione‐S‐transferase (GST), electron transport system (ETS), lactate dehydrogenase (LDH), and cholinesterase (ChE). All analyses were performed in quadruplicate in 96‐well microplates, and absorbance readings were made at 25°C using the microplate reader MultiscanSky.

#### 2.4.2 Protein

Protein quantification was performed according to the Bradford method [22] adapted to microplates and using 4 replicates of each sample. A standard curve was prepared in quadruplicates using bovine *γ*‐globulin with concentrations of 0, 0.2, 0.5, and 1 mg/mL. The dye solution was added to the wells of the microplates and readings were recorded at 600 nm. Protein levels were expressed as mg/mL and mg/g of fresh planarian.

#### 2.4.3 Lipid Peroxidation

Lipid peroxidation (LPO) was determined by measuring thiobarbituric acid reactive substances (TBARS) present in the homogenate samples at 535 nm using 4 replicates per sample [23, 24]. LPO levels were expressed as nmol/g of fresh planarian using a molar extinction coefficient of *ε* = 1.56 × 10^5^ M^−1^ cm^−1^.

#### 2.4.4 Antioxidant Defense System

Catalase (CAT) enzymatic activity was assessed by catalyzing the breakdown of hydrogen peroxide (H2O2) at 240 nm during 3 min using 4 replicates per sample [25], and results were expressed as μmol/min/mg protein using a molar extinction coefficient of *ε* = 40 M^−1^ cm^−1^.

Total glutathione (TG) content was measured by following the formation of a chromogen for 3 min at 412 nm using 4 replicates per sample. The assay was conducted with an excess of glutathione reductase (GR), which recycles oxidized glutathione (GSSG) to reduced glutathione (GSH) after reaction with DTNB (5,5′‐dithiobis‐(2‐nitrobenzoic acid)), and the chromophore formation rate is dependent on total glutathione levels [26, 27]. TG levels were expressed as nmol/mg protein using a standard curve performed with GSH concentrations of 100, 50, 10, and 1 μM.

Glutathione S‐transferase (GST) activity was measured according to the conjugation reaction of reduced glutathione (GSH) with the substrate 1‐chloro‐2,4‐dinitrobenzene at 340 nm for 3 min using 4 replicates per sample [28]. GST activity was expressed as nmol/min/mg protein using a molar extinction coefficient of *ε* = 9.6 × 10^3^ M^−1^ cm^−1^.

#### 2.4.5 Energy Consumption

The electron transport system (ETS) activity assay was performed to estimate energy consumption, involving the addition of electron donors (NADH and NADPH) and an artificial electron acceptor (INT—iodonitrotetrazolium) to the homogenate [29]. Kinetic readings were recorded at 490 nm for 3 min using 4 replicates per sample and used to calculate the oxygen consumption rate (for 2 μmol of INT produced, 1 μmol of oxygen is consumed) by using the molar extinction coefficient of ε = 1.59 × 10^4^ M^−1^ cm^−1^. Results were expressed as nmol O_2_/min/mg protein.

Lactate dehydrogenase activity (LDH) was measured by catalyzing the conversion of pyruvate to lactate using NADH [30]. Kinetic readings were performed at 340 nm for 3 min using 4 replicates per sample. Results were expressed as nmol/min/mg protein using a molar extinction coefficient of *ε* = 6.3 mM^−1^ cm^−1^.

#### 2.4.6 Cholinesterase

Cholinesterase (ChE) activity was measured using acetylthiocholine as substrate at 414 nm using 4 replicates per sample, according to Ellman et al. [31]. ChE activity results were expressed as nmol/min/mg protein.

#### 2.4.7 Calculated Ratios

The LPO/ETS ratio was calculated using LPO levels and ETS activity and expressed as nmol TBARS g^−1^/nmol O_2_ mg^−1^, The ETS/LDH ratio was calculated using ETS and LDH activities and expressed as nmol O_2_/nmol lactate. The ETS/LDH ratio was calculated using ETS and LDH activities and expressed as nmol O_2_/nmol lactate.

### 2.5 Behavioral Biomarkers

Part of the organisms (*n* = 10 per treatment) of the experimental groups that survived the survival tests (12.5%, 25%, 50% and STP effluent, Flona water, and the control using commercial water) were immediately used for post‐exposure locomotor velocity (pLMV) by placing them individually in a 28 cm diameter glass container with commercial water and lined paper grid (0.1 cm spacing) at the bottom [32, 33]. Organisms were placed in the center of the container, and each crossed and recrossed line was counted, and pLMV was determined after 2 min of videorecording. The results were expressed as cm/min.

After the locomotor activity assessment, each planarian was decapitated with a cut behind the auricles using a sterilized scalpel [32, 33]. After decapitation, each planarian was individually placed in a well plate with 10 mL of commercial water. Planarians were individually checked under a stereomicroscope every 12 h post‐decapitation to observe photoreceptor formation until it was complete. The results are presented as the time required to regenerate the photoreceptors in hours.

### 2.6 Statistical Analyses

Data distribution was assessed using the Shapiro–Wilk normality test. Because the experimental design consisted of independent categorical groups (control, reference site water, and different dilutions of treated sewage), differences among treatments were evaluated using one‐way analysis of variance (ANOVA) for parametric data, followed by Tukey's post hoc test for multiple comparisons. When data did not meet the assumptions of normality, the non‐parametric Kruskal–Wallis test was applied, followed by Dunn's post hoc test when significant differences were detected. Statistical analyses were performed using GraphPad Prism software (version 6.0, GraphPad Software Inc., San Diego, CA, USA). Results are expressed as mean ± standard error of the mean (mean ± SEM), and statistical significance was considered at *p* < 0.05.

## 3 Results

### 3.1 Water Samples

The chemical analysis of the water samples showed that the organic elements quantified in the STP effluent never exceed the concentration limits existent in Brazilian legislation (CONAMA 430/2011), although the concentrations observed for iron and phosphorus were higher than the ones accepted in freshwater (Table 2) according to regulatory Brazilian or European limits concerning manganese (CONAMA 365/2005; European Parliament 2020/2184). Moreover, STP effluent also presented high values of magnesium and potassium despite the absence of legislation for their limits in Brazil and Europe (Table 2). It was also evident that STP effluent did not present metals and metalloids in excess when considering the existent legislation considering what is expected in treated effluents (Table 2).

**TABLE 2:** Concentration of inorganic elements (mg/L) in treated sewage (STP) effluent and natural water (Flona) and corresponding regulatory limits established for *effluent discharge in Brazil [34], **freshwater in Brazil [35], and *** water for human consumption in Europe [36].

Inorganic elements (mg/L)	Detection limits	Treated sewage (STP)	Natural water (Flona)	CONAMA 430/2011*	CONAMA 357/2005**	European Parliament 2020/2184***
Arsenic (As)	0.001	0.004	0.005	0.500	0.010	0.010
Cadmium (Cd)	0.001	nd	nd	0.200	0.001	0.005
Chrome (Cr)	0.001	0.011	0.019	1.000	0.050	0.025
Cobalt (Co)	0.001	nd	0.002	—	0.050	—
Copper (Cu)	0.030	nd	0.138	1.000	0.009	2.000
Iron (Fe)	0.086	3.698	13.968	15.000	0.300	0.200
Lead (Pb)	0.001	0.002	0.005	0.500	0.010	0.005
Magnesium (Mg)	0.106	5.683	11.481	—	—	—
Manganese (Mn)	0.002	0.091	0.255	1.000	0.100	0.050
Molybdenum (Mo)	0.001	nd	0.002	—	—	—
Nickel (Ni)	0.001	0.005	0.035	2.000	0.025	0.020
Phosphorus (P)	0.880	9.533	12.322	—	0.100	—
Potassium (K)	0.605	5.791	40.721	—	—	—
Zinc (Zi)	0.026	0.040	1.148	5.000	0.180	—

Abbreviations: nd: non‐detected, below detection limit, ‐: not included in the legislation.

The water from Flona conservation area exhibited concentrations of inorganic metals, including metals and metalloids, exceeding the limit values imposed by legislation imposed by Brazil or Europe for freshwater and drinking water, respectively (Table 2), namely copper, iron, manganese, nickel, and zinc. In addition, Flona water presented high concentrations of magnesium, phosphorus, and potassium (Table 2).

The results of the analysis of pesticides above the detection limit (0.01 ug/L) showed that concentrations of carbendazim, cypermethrin, fipronil, imidacloprid, and pirimifos were above the acceptable limits determined in the 2020/2184 regulation of the European Parliament (Table 3). Brazilian legislation has higher limits of acceptance for some of those pesticides. The water from Flona conservation area did not present any of the pesticides that were determined in this study (Table 3).

**TABLE 3:** Concentration of pesticides (μg/L) detected in treated sewage (STP) effluent and natural water (Flona) and the regulatory limits established for * water used for human consumption in Brazil [37] and ** and in Europe [36].

Pesticides (μg/L)	Detection limits	Treated sewage (STP)	Natural water (Flona)	Ordinance GM/MS No 888/2021*	European Parliament 2020/2184**
Atrazine	0.01	0.08	nd	2	0.1
Azoxystrobin	0.01	0.084	nd	—	0.1
Bifenthrin	0.01	0.028	nd	—	0.1
Carbendazim	0.01	**0.147**	nd	120	0.1
Cypermethrin	0.01	**0.636**	nd	—	0.1
Deltamethrin	0.01	0.08	nd	—	0.1
Diuron	0.01	0.054	nd	20	0.1
Fipronil	0.01	**0.408**	nd	1.2	0.1
Fipronil sulfone	0.01	0.024	nd	—	0.1
Imidacloprid	0.01	**0.211**	nd	—	0.1
Piperonyl butoxide	0.01	0.010	nd	—	0.1
Pirimiphos‐methyl	0.01	**0.104**	nd	—	0.1
Thiamethoxam	0.01	0.056	nd	36	0.1

*Note:* Bold values exceed the limits established for human consumption in Europe.

Abbreviations: nd: non‐detected, below detection limit, ‐: not included in the legislation.

### 3.2 Biomarkers

#### 3.2.1 Body Weight and Protein Content

The body weight (Figure 2A) and protein content (Figure 2B) of planarians were not significantly changed in planarians exposed to treated sewage STP and Flona when compared to control condition.

**FIGURE 2:**
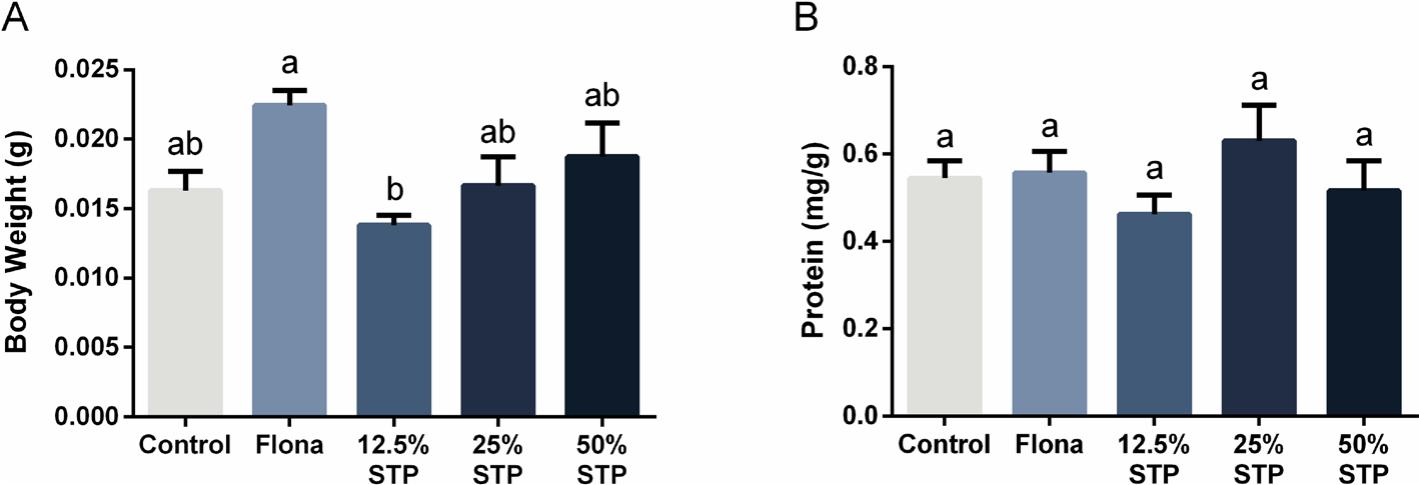
Body weight (A) and protein content (B) in planarians exposed for 48 h to the concentrations of 12.5%, 25%, and 50% of the treated sewage effluent (STP), water from the Silvânia National Forest (Flona) and a control with commercial water of the planarians' culture. The values represent the mean SEM of 10 planarians per treatment. ^a,b^ Different letters indicate a significant difference between groups (*p* < 0.05).

#### 3.2.2 Lipid Peroxidation

The levels of LPO (Figure 3A) and LPO/ETS ratio (Figure 3B) were significantly increased in planarians exposed to 12.5% and 25% STP compared to the control group.

**FIGURE 3:**
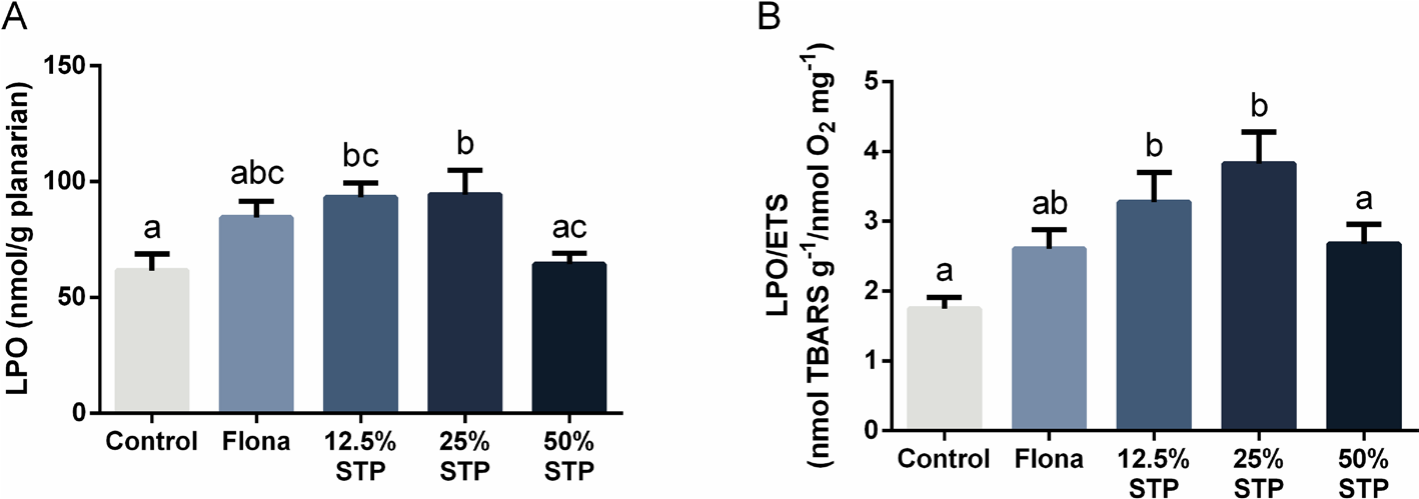
Lipid peroxidation levels (LPO) (A) and the ratio between lipid damage and aerobic energy consumption (LPO/ETS) (B) in planarians exposed for 48 h to the concentrations of 12.5%, 25%, and 50% of the treated sewage effluent (STP), water from the Silvânia National Forest (Flona), and a control with commercial water of the planarians' culture. The values represent the mean SEM of 10 planarians per treatment. ^a,b^ Different letters indicate a significant difference between groups (*p* < 0.05).

#### 3.2.3 Antioxidant Defense and Detoxification Capacities

Catalase enzyme activity was significantly increased in planarians exposed to 25% and 50% STP compared to the control and Flona water groups (Figure 4A). Total glutathione levels, a non‐enzymatic antioxidant, was significantly decreased in planarians exposed to 25% and 50% STP compared to the control and Flona groups (Figure 4B). The activity of glutathione S‐transferase decreased in the 50% STP group compared to the Flona group. (Figure 4C).

**FIGURE 4:**
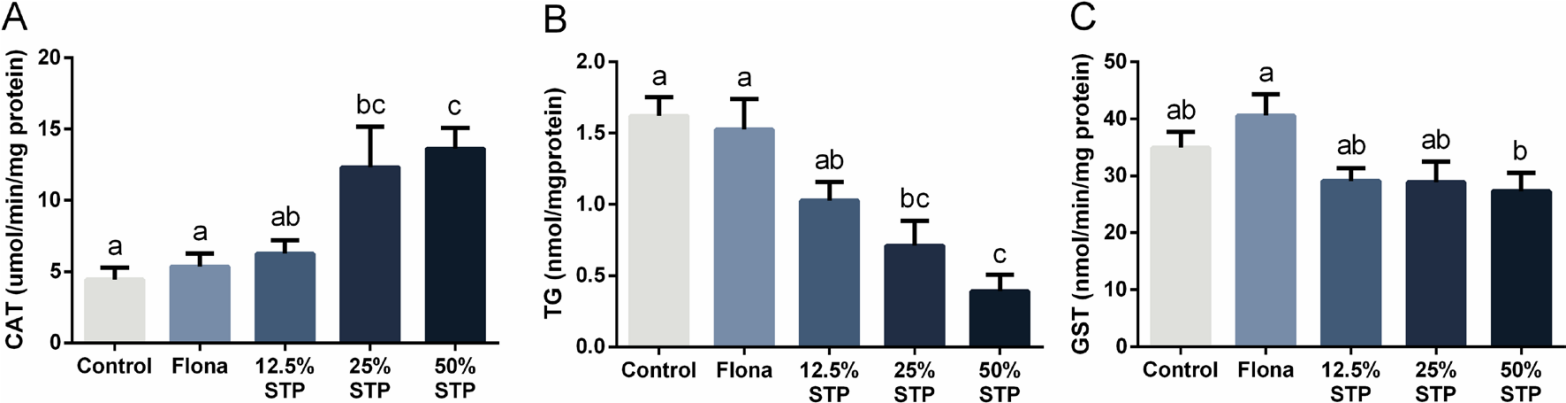
Catalase enzyme activity (CAT) (A), total glutathione levels (TG) (B) and glutathione S‐transferase activity (GST) (C) in planarians exposed for 48 h to the concentrations of 12.5%, 25%, and 50% of the treated sewage effluent (STP), water from the Silvânia National Forest (Flona) and a control with commercial water of the planarians' culture. The values represent the mean SEM of 10 planarians per treatment. ^a,b^ Different letters indicate a significant difference between groups (*p* < 0.05).

#### 3.2.4 Energy Consumption

The activity of ETS was significantly decreased in planarians exposed to 25% and 50% STP compared to the control and Flona groups (Figure 5A), whereas the activity of LDH significantly increased compared to the control and Flona groups (Figure 5B). Thus, the ETS/LDH ratio was significantly decreased in planarians exposed to 25% and 50% STP compared to the Flona group (Figure 5C).

**FIGURE 5:**
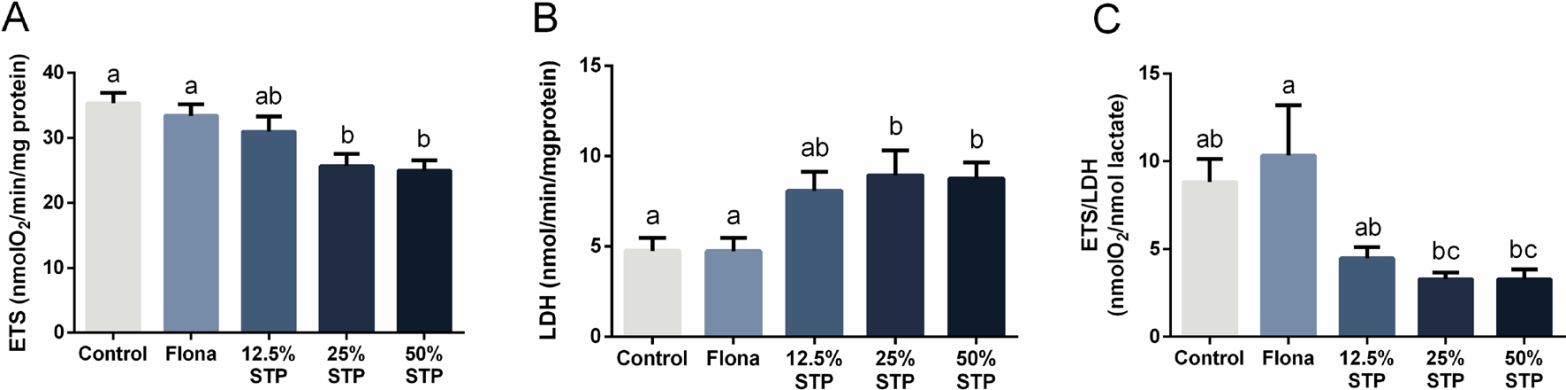
Electron transport system activity (ETS) (A), lactate dehydrogenase activity (LDH) (B) and the ratio between electron transport system activity and lactate dehydrogenase activity (ETS/LDH) (C) in planarians exposed for 48 h to the concentrations of 12.5%, 25%, and 50% of the treated sewage effluent (STP), water from the Silvânia National Forest (Flona) and a control with commercial water of the planarians' culture. The values represent the mean SEM of 10 planarians per treatment. ^a,b^ Different letters indicate a significant difference between groups (*p* < 0.05).

#### 3.2.5 Cholinesterase

There was no difference in cholinesterase activity between the groups (mean ± SEM: Control: 154.6 ± 5.26; Flona: 158.4 ± 7.77; 12.5% STP: 131.8 ± 8.79; 25% STP: 135 ± 9.78 and 50% STP: 123.1 ± 13.86) (Figure S1).

## 4 Behavioral Biomarkers

### 4.1 Locomotor Activity and Regeneration of Photoreceptors

The locomotor activity was significantly decreased in planarians exposed to 50% STP compared to control and Flona water (Figure 6A). The results also show that planarians exposed to Flona water presented a significant increase in the locomotor activity when compared to the control condition (Figure 6A). The time for the regeneration of photoreceptors was increased in planarians exposed to all concentrations of STP compared to the control and Flona groups (Figure 6B).

**FIGURE 6:**
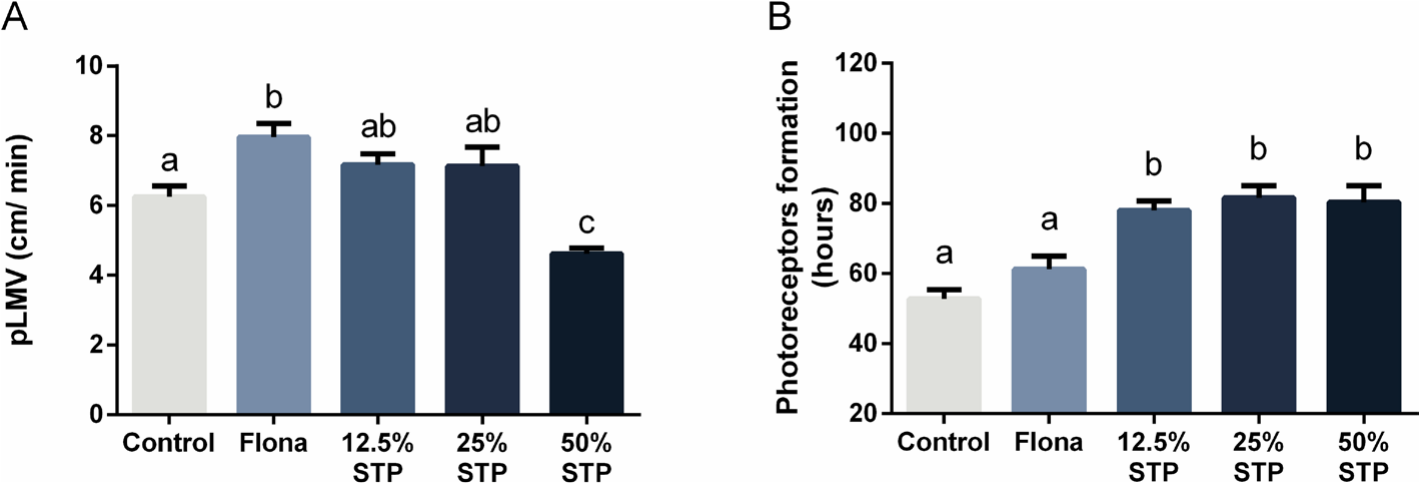
Locomotor activity (A) and regeneration of photoreceptors (B) in planarians exposed for 48 h to the concentrations of 12.5%, 25%, and 50% of the treated sewage effluent (STP), water from the Silvânia National Forest (Flona), and a control with commercial water of the planarians' culture. The values represent the mean SEM of 10 planarians per treatment. ^a,b^ Different letters indicate a significant difference between groups (*p* < 0.05).

## 5 Discussion

The exposure of planarians to the treated sewage resulted in full mortality, highlighting the toxicity of the complex mixture of pollutants that are still present in the treated effluent. After comparison with the water from Flona conservation area, the STP contained a mixture of pesticides, with some of them exceeding the legal levels, namely carbendazim, cypermethrin, fipronil, imidacloprid, and pirimifos. This mixture of pesticides in the treated sewage effluent was above the individual acceptable limits determined in the 2020/2184 regulation of the European Parliament, although considered accepted for the Brazilian legislation. Interestingly, even after laboratory dilution, the treated sewage induced oxidative stress responses in the non‐target planarians. Nevertheless, current results considering the oxidative stress induced by the treated sewage do not exclude the possibility that other pollutants not quantified could also be synergistically contributing to the observed oxidative damage due to the presence of the 5 pesticides, but not the elements including metals and metalloids, since Flona water did not alter the oxidative condition of planarians. Although some elements exceeded regulatory limits at the Flona site, these concentrations are likely related to local soil geochemistry and surrounding agricultural activities [9, 38, 39] and did not influence planarian responses, as biomarker levels were comparable to those observed in the control group.

Although planarians belong to a group of invertebrates that may exhibit a relatively higher tolerance to organic pollution when compared to more sensitive taxa such as Ephemeroptera, Plecoptera, and Trichoptera [14, 40, 41], they proved to be effective bioindicators for detecting sublethal and mechanistic responses to treated sewage. A previous study using water from an urban river also reported oxidative damage in planarians, even with metal concentrations below governmental limits [17]. In our current study, the oxidative stress condition of planarians seems to be associated with the increased levels of lipid peroxidation concomitant with decreased nonenzymatic antioxidant capacity. Moreover, the increased enzymatic antioxidant capacity observed as increased catalase activity does not seem sufficient to avoid oxidative damage to lipids due to the excess production of reactive oxygen species.

High levels of LPO were observed in planarians exposed to 12.5% and 25% of the treated sewage, followed by a reduction when exposed to 50%. The absence of LPO in planarians might be due to the decreased aerobic production of energy that consequently diminishes the levels of reactive oxygen species. Therefore, the activation of lactate dehydrogenase as a backup compensatory energetic alternative is an indication of an extreme stress condition for the highest concentration of STP. This is significantly evident in planarians exposed to 50% of the treated sewage when considering the ETS/LDH and LPO/ETS ratios. Additionally, the decreased tendency of GST activity for planarians exposed to 50% of treated sewage might also be due to damage to protein and/or severe depletion of reduced glutathione, one substrate of the enzyme.

The decreased levels of total glutathione across all groups of planarians exposed to dilutions of the treated sewage show that initially, there was an attempt to mitigate reactive oxygen species (ROS) through non–enzymatic antioxidants that were insufficient and therefore an activation of CAT activity occurred. Moreover, a metabolic shift in the acquisition of energy occurred from aerobic production to anaerobic, which helps to diminish oxidative stress apparently. Nevertheless, it cannot be excluded that the reduction of LPO levels observed in planarians exposed to 50% might also be due to the depletion of available lipids for oxidation or the triggering of cellular apoptosis at a critical level of oxidative stress [42]. Similar results were reported in planarians exposed to polycyclic aromatic hydrocarbons (PAHs), where a reduction in LPO was observed without a compensatory antioxidant response [43]. Therefore, chronic exposure experiments are warranted and are proposed as a future research direction to determine whether the LPO response reflects adaptive physiological adjustments or the onset of delayed toxic effects or a near death condition associated with apoptosis as mentioned above.

The reduction in GST and TG levels in the exposed groups reinforces the hypothesis that the detoxification mechanisms and glutathione regeneration pathways were overwhelmed, rendering them unable to adequately respond to the increased ROS load generated by higher concentrations of treated sewage. In addition to lipid damage, ROS generation and the consequent depletion or inactivation of the antioxidant system can lead to DNA damage and enzymatic inhibition, reflecting the damage to proteins. Prá et al. [15] demonstrated that genotoxicity in planarians is associated with high levels of pollution, eutrophication, and the urbanization gradient of the studied river. These findings underscore the complexity of the toxic effects of pollutants and the need for further studies on aquatic organisms.

The observed alterations in energy consumption suggest a state of extreme metabolic stress in planarians, characterized by a reduction in aerobic respiration and an increase in anaerobic metabolism. Although Mg, K, and P were not the primary focus of this study on oxidative damage, nutrient enrichment from wastewater discharges may influence aquatic ecosystems by stimulating microbial activity and organic matter decomposition, processes that can reduce dissolved oxygen availability and potentially affect aquatic bioindicators. However, it is important to note that the concentrations of these nutrients were also high in the Flona reference area, where planarians showed biomarker responses comparable to those observed in the control group. This suggests that the oxidative and metabolic alterations observed in organisms exposed to the STP effluent are more likely associated with other contaminants present in the treated sewage. Pollutants such as metals, pesticides, and pharmaceuticals are known to interact directly or indirectly with mitochondria, often through the generation of ROS. These interactions can lead to dysfunction in the electron transport chain [44, 45], resulting in reduced aerobic energy production. Consequently, planarians were forced to rely on anaerobic metabolism to meet their energy demands, but also diminishing the levels of reactive oxygen species to prevent more oxidative damage.

Therefore, the plausible explanation for the observed changes in energy metabolism could be a short‐term adaptive strategy to reduce intracellular oxygen availability, thereby decreasing ROS generation and mitigating oxidative damage. Studies on mollusks exposed to pharmaceuticals support this hypothesis, showing reduced ETS activity at higher concentrations [46]. Interestingly, lipid damage was observed at lower concentrations and decreased at higher concentrations [46], consistent with our findings. These results suggest that higher ETS activity is directly associated with increased ROS production, as mitochondria are the primary site of ROS generation during respiration. The increase in the LPO/ETS ratio in the 12.5% and 25% treated sewage‐exposed groups suggests that lipid peroxidation is affecting mitochondrial function and impairing ATP production. Conversely, the decrease in this ratio in the 50% group is associated with critical stress, as previously mentioned.

The increase in LDH activity under stress conditions in planarians appears to be a critical response, ensuring sufficient energy supply to cope with other toxic effects. Similar results have been reported in studies on snails exposed to pollutants such as metals and pesticides, where anaerobic metabolic pathways were activated as compensatory mechanisms [47]. Conversely, in planarians exposed to flame retardants and microplastics, neither individual nor combined exposure resulted in increased LDH activity [48]. Although aerobic metabolism remains functional, the decrease in the ETS/LDH ratio may indicate a shift from aerobic to anaerobic metabolism as stress intensifies.

A reduction in ETS activity in resource‐limited natural environments is expected to further restrict energy allocation to vital functions and negatively affect organismal fitness. All efforts aimed at mitigating oxidative damage and metabolic alterations appear to have compromised fundamental processes such as locomotion and regeneration in planarians exposed to treated sewage. Moreover, pollutants present in the effluent may induce behavioral changes in these organisms. The observed reduction in locomotion speed and delayed regeneration time may be attributed to damage in the neuromuscular system, potentially caused by excessive ROS production and lipid peroxidation [49]. Although our results did not reveal significant differences in cholinesterase levels among the tested groups, this suggests that, while acetylcholinesterase (AChE) activity was not directly inhibited, other neurological processes may have been affected. Damage to cell membranes or neural structures could have contributed to the locomotor impairments observed. Previous studies on planarians exposed to metals and neurotoxic compounds such as flame retardants, PAHs, and pesticides also reported locomotion alterations. These findings highlight locomotion as a sensitive biomarker for assessing environmental toxicity [48, 50, 51, 52].

The delayed regeneration of photoreceptors in groups exposed to treated sewage may be associated with impaired cellular division and differentiation processes [53]. Previous research on nano and microplastics [33, 54], cadmium and copper [55], neonicotinoids [56], and drugs [57] also reported delays or alterations in regeneration patterns in exposed planarians. Since regeneration in planarians heavily relies on the activity of pluripotent stem cells, exposure to effluents containing complex mixtures of pollutants could severely impair this regenerative capacity. This has significant implications for the survival of planarians in contaminated environments, potentially impacting ecological stability and recovery processes in polluted aquatic habitats.

A previous study by our group on planarian aging observed that older individuals (9–12 months) exhibited increased lipid peroxidation (LPO) levels associated with reduced aerobic energy production and diminished antioxidant capacity, ultimately compromising vital functions. Our current results suggest that planarians from the control group and those exposed to water from the conservation area maintain a biological age consistent with their chronological age (2 to 6 months). In contrast, planarians exposed to the treated sewage (STP) showed some signs of accelerated biological aging considering the impairment of locomotor activity, decreased aerobic production of energy, and levels of TG. However, exposed planarians kept an increased CAT activity and triggered LDH, which was not observed in aged planarians. Therefore, those signs of aging in planarians exposed to STP might be transient, and further studies concerning the analysis of biomarkers after recovery of STP exposed planarians would be important.

## 6 Conclusions

This study demonstrated that exposure of planarians to different concentrations of treated sewage induces biochemical, energetic, and behavioral disturbances. Despite metal and pesticide concentrations being below Brazilian regulatory limits, the results indicate that complex mixtures of pesticides present in treated effluents may exacerbate toxicity through synergistic or additive effects.

The evaluated biomarkers revealed that exposed organisms experienced oxidative stress due to reduced non‐enzymatic antioxidants and detoxification capacities. The increase in enzymatic antioxidant activity was insufficient to prevent oxidative damage, except following a metabolic shift related to energy acquisition. Energy depletion was also associated with reduced locomotor activity and impaired regeneration, suggesting neurofunctional impairment effects despite the absence of alterations in the cholinergic system.

These findings highlight the importance of revising effluent regulatory parameters and improving wastewater treatment technologies to mitigate impacts on aquatic ecosystems, particularly considering the differential tolerance of aquatic invertebrate groups to wastewater discharges. Future studies should aim to identify specific compounds responsible for the observed effects and to assess long‐term consequences in planarians and other aquatic organisms with varying sensitivity levels.

## Author Contributions

**Renata M. P. Freitas:** conceptualization, data curation, formal analysis, investigation, methodology, and writing – original draft and writing – review and editing. **Pedro H. Carvalho:** methodology, and writing – review and editing. **Daniela de Melo e Silva:** conceptualization, funding acquisition, resources, supervision, validation, and writing – review and editing. **Carlos Gravato:** conceptualization, funding acquisition, resources, supervision, validation, and writing – review and editing.

## Funding

Coordination for the Improvement of Higher Education Personnel (CAPES, no 933660/2024‐01) and Foundation for Science and Technology (FCT) (Portugal).

## Conflicts of Interest

The authors declare no conflicts of interest.

## Supporting information


**Figure S1:** Cholinesterase activity in planarians exposed for 48 h to the concentrations of 12.5%, 25%, and 50% of the treated sewage effluent (STP), water from the Silvânia National Forest (Flona) and a control with commercial water of the planarians' culture. The values represent the mean SEM of 10 planarians per treatment. ^a,b^ Different letters indicate a significant difference between groups (*p* < 0.05).

## Data Availability

The data that support the findings of this study are available from the corresponding author upon reasonable request.
