# Supplementary material for: Oxidative Damage, Energy Imbalance, and Behavioral Disruptions in Girardia tigrina Exposed to Treated Sewage Effluents
Source: Environ Toxicol. 2026 Apr 8;41(12):1646–57. doi: 10.1002/tox.70098 (PMC13749300; doi:10.1002/tox.70098)
Supplement: Supplementary file 1 [file TOX-41-1646-s001.docx]

**Oxidative damage, energy imbalance, and behavioral disruptions in *Girardia tigrina* exposed to treated sewage effluents**

Renata M. P. Freitas^a,c*^, Pedro H. Carvalho^a,d^, Daniela de Melo e Silva^c^, Carlos Gravato^a,b^

**Supplemmentary Material**

**Figure S1.** Cholinesterase activity in planarians exposed for 48 hours to the concentrations of 12.5, 25, and 50 % of the treated sewage effluent (STP), water from the Silvânia National Forest (Flona) and a control with commercial water of the planarians’ culture. The values represent the mean SEM of 10 planarians per treatment. ^a,b^ Different letters indicate a significant difference between groups (p < 0.05).
